# Renal Medicine

**Published:** 1895-12-28

**Authors:** 


### RENAL MEDICINE.

Infectious nephritis is, though rare, not unknown, and may prove the starting point for disease of a more ordinary type. Duncan Burgess[1] discusses the symptoms seen during an outbreak in which five members of a family were attacked, and three died. Some of the phenomena were those of acute, and others those usually seen in chronic, types of the disease. Uræmia, hæmaturia, and albuminuric retinitis were noticed. The cases followed each other within a few months, but there was no evidence of scarlet fever or cold. A toxic origin for Bright's disease is already recognised in lead poisoning, syphilis, and the nephritis of fevers. The influence of cold is, Burgess thinks, exaggerated, that of alcohol not definitely made out; while probably more cases are due to toxins than is usually thought. Another occasional occurrence is the origin of granular contracted kidney from acute nephritis, as in a case given by T. Dixon Mann.[2] Fürbringer remarks that acute nephritis either is cured or ends fatally unaltered, but in rare cases it may pass over into another type. Mann's patient had scarlet fever, and the albuminuria continued afterwards under his observation for 28 years. Sub-acute attacks at first occurred, and finally the type of disease changed, the urine became copious and of low gravity, the arterial tension rose, and after death typical granular kidneys were found, with great increase of the connective tissue throughout the cortex. Similar cases have been reported by Leyden and others.

Pyrexia is not altogether unknown in Bright's disease. There is frequently seen fever in acute nephritis, which may be severe in the infectious cases above mentioned; then there is a form due to complications, such as pleuritis; and, lastly, pyrexia may sometimes accompany uræmia. Stengel[3] points out that this may either be sudden and pronounced, accompanied, too, with coma and convulsions, or more general and lasting, associated with typhoid symptoms. The fever cannot be due to the convulsions in the uræmic form, for it often precedes them by some days. It appears to be a comparatively rare occurrence, and is probably caused by the absorption of some pyrogenous substance from the urine. Krehl and Matthes[4] have investigated the causes of this pyrexia, but found no method of estimating the action in this respect of assimilable albumens. However, they show that hydrated albumens or albumoses are usually present in the urine during pyrexia, and rarely exist when the temperature is normal, and, finally, that these bodies belong to the class of deutero-albumoses.

The importance of hæmato-porphyrin in urine was discussed by Garrod and Hopkins[5] in a recent paper. There are traces in normal urine, but the larger amounts in fatal cases have usually followed the taking of sulphonal. No previous ill-effects from the drug have usually been noticed until abdominal pain and collapse, with dark coloured urine, appear. Alkaline treatment seems to have been of use in some of these cases. Stokvis gave sulphonal[6] to rabbits and dogs, and found that hæmato porphyrin then made its appearance in the urine, sometimes with urobilin. Hæmorrhages, too, were seen post-mortem in the mucous membrane of the stomach, and some of the absorbed porphyrin is retained in the liver.

Most probably the sulphonal acts as a corrosive poison after passing through some change in the body. Lafon found a reducing substance[7] in the urine of a patient taking sulphonal daily, which did not give the right-handed deviation in the polariscope usually found with diabetic sugar. These results from sulphonal, when given frequently or for long periods, indicate the need of great watchfulness in its administration. Much is doubtless due to idiosyncrasy, as in the case of quinine,[8] which occasionally in malarial subjects causes severe hæmaturia. Tomaselli warns us against giving quinine when this tendency exists. Further researches on the importance of indican in the urine point to its being a result of intestinal putrefaction or absorption of pus. Simon[9] regards it as an indication of the amount of free hydrochloric acid present, except where it is associated with ulcer of the stomach. Otherwise, where hydrochloric acid is normal or in excess, there is never excessive elimination of indican. On the question of albuminuria Richter insists[10] that a purely physiological form exists, because, among other reasons, it is found so frequently and out of all proportion to the incidence of renal disease. It is impossible to believe that anything like the number of healthy soldiers in whom it is found tend to become subjects of renal disease later on. He insists, however, that cases must be excluded where the urine varies in amount and corpuscular elements are present, and also those where the excretion of albumen is persistent, large in amount, or the patient past middle age or showing signs of ill-health. Simon considers the evidence for purely physiological excretion as distinct from functional albuminuria to be defective. It usually indicates some abnormal action of some part of the body, and an important form is seen in the digestive albuminuria of Da Corta, where nerve symptoms with high specific gravity of the urine, and an increase of urea, uric acid, and oxalic acid may often be found. These symptoms disappear under a rigid diet for a time, of which milk forms a large part. On the whole this functional albuminuria has little importance with respect to the kidneys, though it may point to a weakness elsewhere in the organism. Sir Richard Quain,[11] in an amusing address recently, laid stress on the value of mercury in albuminuria under certain circumstances. It has been decried as dangerous since the days of Dr. Blackwall, of Exeter, a contemporary of Bright, but Quain related several instances of its value even when its use was long continued. The instances seem to be chiefly those where digestive and liver troubles were prominent, but occasionally it exerts a beneficial effect on the œdema of renal disease and the excretion of albumen. Huchard speaks highly of his experience with theobromine.[12] It has no ill-effects on the nervous, circulatory, or digestive systems, nor on the kidneys. Its diuretic action continues longer than that of caffeine, but for a shorter time than digitalis, and the amount of water excreted is immense. Diuretin and the compounds of theobromine he regards as untrustworthy. Lithium bromide[13] is similarly praised by Polakow, especially in acute nephritis. He found it a powerful diuretic, increasing the urine, and diminishing the albumen and œdema. The good effects appeared in about a fortnight at the latest, and if a break was made in the administration of the salt after improvement had ceased, and it was then again given, further beneficial results followed. Some two grammes of the bromide, with four of sodium bicarbonate, were mixed in 240 grammes of distilled water, and three tablespoonfuls were given in the day. Hirshfield,[14] in discussing the value of diet in albuminuria, argues that the loss of albumen cannot be made up by increased proteid diet, for this increases metabolism as well as the amount of extractives to be got rid of. In opposition to the doctrine of Hale White, he believes that a rich proteid diet will increase the tendency to uræmia, for the increased excretion of extractives on this diet occurs later in patients with diseased kidneys than under normal conditions. He gives in chronic Bright's disease a mixed diet with a large amount of carbohydrates. Milk may be given alone in small quantities in acute Bright's disease, or alone in large amounts—six or eight pints daily—for a time, as recommended by Germain See, or merely in addition to the usual mixed diet. Urotropin has been found useful by Professor A. Nicolaier[15] against uræmia and in causing uric acid gravel to disappear. It also prevents ammoniacal fermentation in cystitis. He recommends doses of fifteen to twenty grains a day. Though many persons bear larger doses without pain, in some cases large doses cause troublesome irritation.

[1] Quarterly Med. J., Oct. [2] Lancet, Sept. 14. [3] Am. J. Med. Sc., Nov. [4] Am. M. S. Bulletin, Oct. 1. [5] B. M. J., Nov 4. [6] Am. J. Med. Sc, Oct. [7] B. M. J., Sept. 14. [8] B. M. J., Sept. 14. [9] Med. Chronicle, Sept. [10] Am M. S. Bulletin, Sept. 15. [11] B. M. J., Nov. 9. [12] Am. J. Med. Sc., Oct. [1] Med. Week, Sept. 13. [14] Practit., Sept. [15] Med. Week., Sept. 20.

